# Multiscale Compressive Failure Analysis of Wrinkled Laminates Based on Multiaxial Damage Model

**DOI:** 10.3390/ma18194503

**Published:** 2025-09-27

**Authors:** Jian Shi, Guang Yang, Nan Sun, Jie Zheng, Jingjing Qian, Wenjia Wang, Kun Song

**Affiliations:** 1 College of Aviation Engineering, Civil Aviation Flight University of China, Chengdu 641419, China; shijian@cafuc.edu.cn; 2 School of Fiber Engineering and Equipment Technology, Jiangnan University, Wuxi 214000, China; 3 Department of Mechanical Engineering, University of Alberta, Edmonton, AB T6G 2R3, Canada; jzheng11@ualberta.ca; 4 Department of Electrical and Computer Engineering, University of Alberta, Edmonton, AB T6G 2V4, Canada; sunnan@nefu.edu.cn; 5 Research Institute of Science and Technology Innovation, Civil Aviation University of China, Tianjin 300300, China; jjqian@cauc.edu.cn; 6 School of Computer and Data Science, University of Hong Kong, Hong Kong 999077, China; wwj2022@connect.hku.hk; 7 School of Mechanical and Power Engineering, Nanjing Tech University, Nanjing 211816, China

**Keywords:** wrinkle defects, multiscale analysis, generalized method of cells, multiaxial progressive damage model

## Abstract

The waviness defect, a common manufacturing flaw in composite structures, can significantly impact the mechanical performance. This study investigates the effects of wrinkles on the ultimate load and failure modes of two Carbon Fiber Reinforced Composite (CFRC) laminates through compressive experiments and simulation analyses. The laminates have stacking sequences of [0]_10S_ and [45/0/−45/90/45/0/−45/0/45/0]_S_. Each laminate includes four different waviness ratios (the ratio of wrinkle amplitude to laminate thickness) of 0%, 10%, 20% and 30%. In the simulation, a novel multiaxial progressive damage model is implemented via the user material (UMAT) subroutine to predict the compressive failure behavior of wrinkled composite laminates. This multiscale analysis framework innovatively features a 7 × 7 generalized method of cells coupled with stress-based multiaxial Hashin failure criteria to accurately analyze the impact of wrinkle defects on structural performance and efficiently transfer macro-microscopic damage variables. When any microscopic subcell within the representative unit cell (RUC) satisfies a failure criterion, its stiffness matrix is reduced to a nominal value, and the corresponding failure modes are tracked through state variables. When more than 50% fiber subcells fail in the fiber direction or more than 50% matrix subcells fail in the transverse or thickness direction, it indicates that the RUC has experienced the corresponding failure modes, which are the tensile or compressive failure of fibers, matrix, or delamination in the three axial directions. This multiscale model accurately predicted the load–displacement curves and failure modes of wrinkled composites under compressive load, showing good agreement with experimental results. The analysis results indicate that wrinkle defects can reduce the ultimate load-carrying capacity and promote local buckling deformation at the wrinkled region, leading to changes in damage distribution and failure modes.

## 1. Introduction

Composite structures exhibit anisotropic characteristics, having complex and variable microstructural morphologies. Many defects are inevitably formed during the manufacturing process, and fiber waviness is one of the most common defects. The causes of waviness defects are diverse, such as residual stress, uneven curing, spatiotemporal gradient differences in temperature fields, and processing parameters. Common waviness defects include out-of-plane waviness and in-plane waviness [1]. Early studies mostly focused on in-plane waviness, which has a more severe impact on the mechanical properties of composite structures [2,3]. However, as the excellent properties of composite materials continue to be explored, they are widely applied in large-scale thick-section structural components. Out-of-plane waviness can significantly reduce the ultimate load of composite structures and lead to complex stresses and damage states, resulting in premature structural failure [4,5,6]. The failure analysis of defective composite laminates is critical in structural design, so it is of great importance to research the influence of out-of-plane wrinkling defects on composite structures. Under compressive loading, common failure mechanisms observed in wrinkled laminates include matrix cracking, fiber fracture, and delamination [7,8,9]. Nimbal et al. [10] studied the influence of fiber wrinkling parameters on compressive properties, and the results showed that parameters such as the maximum wrinkling angle and the area of resin accumulation zones all affect the compressive strength. Recent studies have found that the dominant failure modes of composite laminates are closely related to the degree of fiber wrinkling. Mukhopadhyay et al. [11] used a combination of experimental and numerical simulation methods to study the compressive failure mechanism of laminates with fiber wrinkling. They found that the dominant failure mode transitions as the wrinkling angle increases. Davidson et al. [12] investigated the effects of fiber waviness misalignment angle and wrinkle amplitude on the compressive strength of carbon fiber composites through experiments and finite element methods. The results showed that both variables significantly reduce the compressive strength of the material, and the final failure mode is mainly affected by the aspect ratio. Reference [13] studied the compressive strength of wrinkled composites and pointed out that the strength of specimens with wrinkles is significantly lower than that of specimens without wrinkles.

Analytical and numerical models have been developed to predict the failure mechanisms of composite laminates with wrinkle defects [14]. Macroscopic failure criteria are often used to determine the initiation of structural failure [15,16], with some failure models validated in the World-Wide Failure Exercise (WWFEs) [17,18]. Although many criteria distinguish between fiber and matrix failures and incorporate progressive damage models [19,20], they cannot capture the interactions between constituents at the microscopic scale, making it difficult to elucidate the mechanisms of damage initiation and propagation at the microstructural level. Thus, using micromechanics to characterize the damage state of composite structures is of significant importance. Micromechanical approaches can account for variations in fiber volume fraction, constituent material properties, or microstructures, enabling a better understanding of failure mechanisms in composite structures. Additionally, these methods easily quantify the interactions between different constituents in composites.

The Generalized Method of Cells (GMC), first proposed by Paley and Aboudi [21], was later improved by taking microscopic stresses as basic unknowns to enhance computational efficiency [22,23,24,25]. Being inherently analytical, this method has been proven to be a highly effective and efficient micromechanical approach. The rectangular repeating unit cell (RUC) is divided into a specified number of rectangular subcells, each assigned a specific constituent material to compute the properties of composite laminates. GMC employs homogenization methods, enforcing displacement and traction continuity conditions across subcell interfaces and RUC periodic boundaries in an average sense. Based on these continuity conditions, a stress concentration matrix linking subcell stresses to RUC strains is established. Utilizing the constitutive equations, subcell stresses can be expressed in terms of RUC (macroscopic) strains. Finally, homogenization is applied to obtain the overall stiffness of the RUC. Embedding the GMC microscopic model into the integration points of finite element models enables robust multiscale analysis, facilitating the investigation of damage initiation and failure mechanisms in multiscale frameworks.

This study investigates the effects of different wrinkle amplitude-to-thickness ratios on the failure modes and ultimate loads of laminates through compressive tests and multiscale progressive damage analysis. The multiscale framework is based on a 7 × 7-scale GMC repeating unit cell, incorporating a multiaxial progressive damage algorithm at the constituent material level (subcells). Initial failure of each subcell is determined using the multiaxial Hashin failure criterion, with corresponding material properties degraded according to the multiaxial failure state of the subcells. To enhance computational efficiency due to the large number of micro-scale damage variables, this study innovatively considers the RUC to exhibit a specific failure mode when 50% of the subcells in a given orientation satisfy the failure criteria. Multiscale analyses are performed in Abaqus 2022 finite element software, using a user material subroutine (UMAT) to implement the GMC model and multiscale damage algorithm. State variables were employed to record, transfer, and update the failure states of subcells and RUC throughout the analysis.

## 2. Multiaxial Mixed Mode Multiscale Model

**Figure 1:**
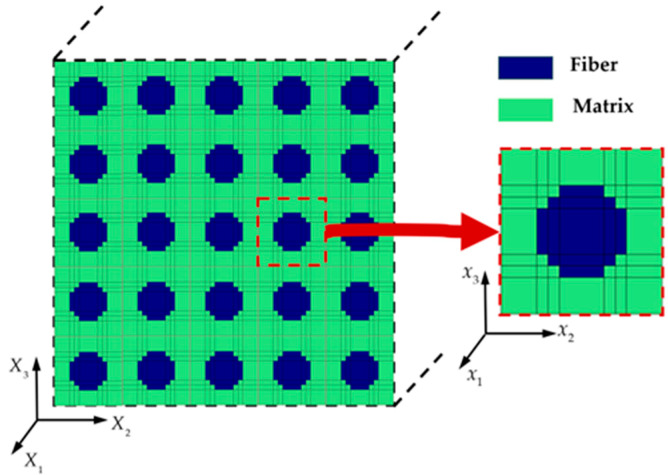
7 × 7 architecture of the generalized method of cells.

The square-packed RUC consisting of 36 matrix subcells (colored green) and 13 fiber subcells (colored blue) is chosen to analyze the macroscopic properties of composite materials. Figure 1 illustrates the GMC model in the microscopic coordinate system (*x*_1_, *x*_2_, *x*_3_), by applying periodic boundary conditions to the RUC's edges and homogenizing the stiffness matrices of all subcells, the effective material properties of the lamina in the macroscopic coordinate system (*X*_1_, *X*_2_, *X*_3_) can be characterized.

Failure criteria are applied to constituent materials, i.e., each subcell within the RUC. Damage initiation in each subcell is determined using the 3D extension of the stress-based Hashin criterion [26,27], as detailed in Equations (1)–(4).

(1) $${\left(\frac{\sigma_{11}}{X}\right)}^{2}+{\left(\frac{\sigma_{12}}{S_{12}}\right)}^{2}+{\left(\frac{\sigma_{13}}{S_{13}}\right)}^{2}\geq 1$$

(2) $${\left(\frac{\sigma_{22}}{Y}\right)}^{2}+{\left(\frac{\sigma_{12}}{S_{12}}\right)}^{2}+{\left(\frac{\sigma_{23}}{S_{23}}\right)}^{2}\geq 1$$

(3) $${\left(\frac{\sigma_{33}}{Z}\right)}^{2}+{\left(\frac{\sigma_{13}}{S_{13}}\right)}^{2}+{\left(\frac{\sigma_{23}}{S_{23}}\right)}^{2}\geq 1$$

(4) $$X=\left\{\begin{array}{l} X_{T}\;\sigma_{11}>0\\ X_{C}\;\sigma_{11}<0 \end{array}\right\},\;Y=\left\{\begin{array}{l} Y_{T}\;\sigma_{22}>0\\ Y_{C}\;\sigma_{22}<0 \end{array}\right\},\;Z=\left\{\begin{array}{l} Z_{T}\;\sigma_{33}>0\\ Z_{C}\;\sigma_{33}<0 \end{array}\right\}$$

where $\sigma_{11},\;\sigma_{22}$ and $\sigma_{33}$ are the normal stress components, *X_T_* and *X_C_* represent the tensile and compressive strengths in the *x_1_*-coordinate direction, *Y_T_* and *Y_C_* denote the tensile and compressive strengths in the *x_2_*-coordinate direction, *Z_T_* and *Z_C_* are the tensile and compressive strengths in the *x_3_*-coordinate direction, and *S_ij_* (*i*, *j* = 1, 2, 3, *i* ≠ *j*) correspond to the shear strengths associated with shear stress components $\sigma_{ij}$.

Damage initiation is triggered when any of the specified failure criteria (Equations (1)–(3)) are satisfied, accompanied by a reduction in the elastic modulus of the failed subcells. Notably, although the fiber phase is transversely isotropic and the matrix phase is isotropic prior to damage, damaged constituents are modeled as orthotropic materials to characterize the multiaxial stiffness degradation. Under compressive loading, the fiber subcells within the 0° plies serve as the primary load-bearing constituent and play a critical role in determining the ultimate compressive strength. Concurrently, the fibers exhibit characteristic brittle fracture behavior. So, a sudden degradation of material property rules, as detailed in Table 1, is employed to reduce the moduli of failed subcells. To prevent stiffness matrix singularity during computations, the degradation coefficient is assigned a small value of 10^−4^, ensuring numerical stability while capturing the abrupt loss of load-carrying capacity.

At each elemental material point, a micromechanical analysis is performed using the GMC by calling the UMAT subroutine, in which multiaxial Hashin failure criteria are implemented to predict subcell failure. Finite element analysis (FEA) obtains the global strain tensors of every element integration point, which are imposed as basic known variables on the RUC to calculate the global stress at each iteration based on GMC. Additionally, the degradation of constituent material properties based on failure criteria reduces the effective stiffness of the RUC, enabling the implementation of the multiscale progressive damage framework.

Due to the geometric symmetry of the 7 × 7-scale RUC, failure at the macroscopic scale is considered to occur when the number of failed subcells in a specific direction exceeds 50% of the total subcells in that orientation. This threshold triggers the corresponding macroscopic failure mode in the finite element. Failure states are tracked using solution-dependent state variables (SDVs) within the Abaqus 2022 UMAT subroutine. The SDV array contains 300 variables (6 SDVs multiply the number of RUC and subcells), where SDV1 to SDV6 represent tensile and compressive failures in the three principal directions of the RUC. The remaining SDVs record tensile and compressive failure states of individual subcells in three axial directions. An SDV value of 0 indicates no failure, while 1 signifies failure initiation.

**Figure 2:**
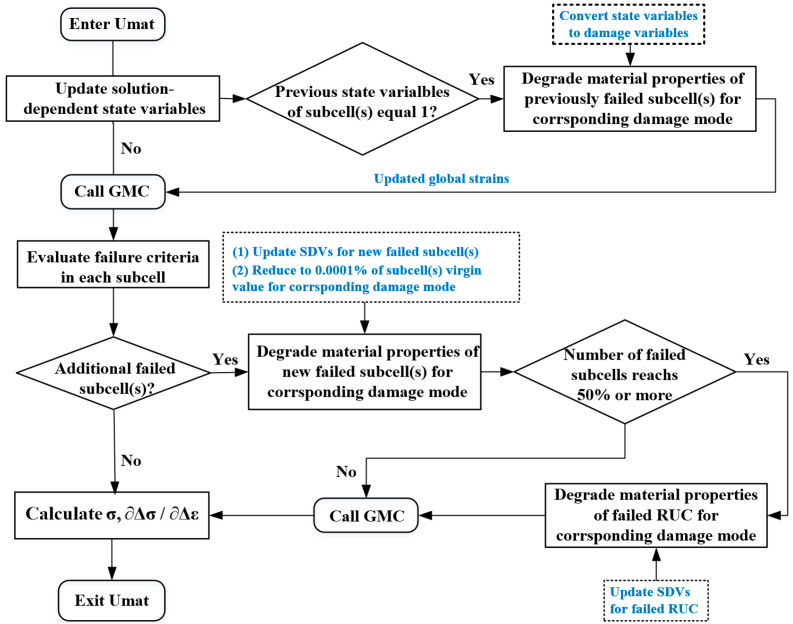
Flowchart of multiscale failure analysis using GMC.

Figure 2 illustrates the flowchart of the GMC-based multiscale failure analysis. Upon entering the UMAT subroutine, the program first updates the subcell stiffness matrices based on the damage states stored in the SDVs. It then applies the updated macroscopic strain from FEA, executes the GMC algorithm to compute subcell stresses, and evaluates new failures against the criteria. If any new subcell fails, it is recorded using SDVs, and its material properties are reduced according to the corresponding failure mode. The program subsequently checks if the failed subcell count of per failure mode exceeds 50%. If satisfied, the RUC-level failure flags are updated. The analysis proceeds iteratively until convergence is achieved.

## 3. Compressive Experiments of Winkled Composite Laminates

**Figure 3:**
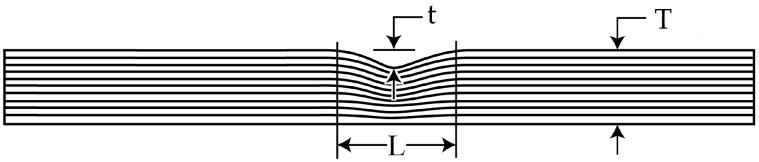
Geometric dimensions of wrinkled specimens.

Eight specimens of two laminates are prepared for different amplitude-to-thickness ratios (denoted as *t*/*T*), as shown in Figure 3. The specimens measure 140 mm in length and 12 mm in width, comprising 20 plies with a single-ply thickness of 0.19 mm and a total thickness *T* of 3.8 mm. The first laminate (Laminate 1) is a unidirectional 0° composite, and a symmetric layup sequence of the second laminate (Laminate 2) is [45/0/−45/90/45/0/−45/0/45/0]_s_, both laminates have a wrinkle span *L* of 5.5 mm. The wrinkle amplitude *t* is set at 0%, 10%, 20%, and 30% of the total thickness *T*, corresponding to 0mm, 0.38 mm, 0.76 mm, and 1.14 mm, respectively.

Uniaxial compression experiments on wrinkled specimens are conducted following ASTM D6641 [28], that is the standard test method for compressive properties of polymer-matrix composite laminates. Tests are performed using an electromechanical universal testing machine, where specimens are clamped at both ends by fixtures and subjected to a compressive displacement load at a rate of 1 mm/min. 

**Figure 4:**
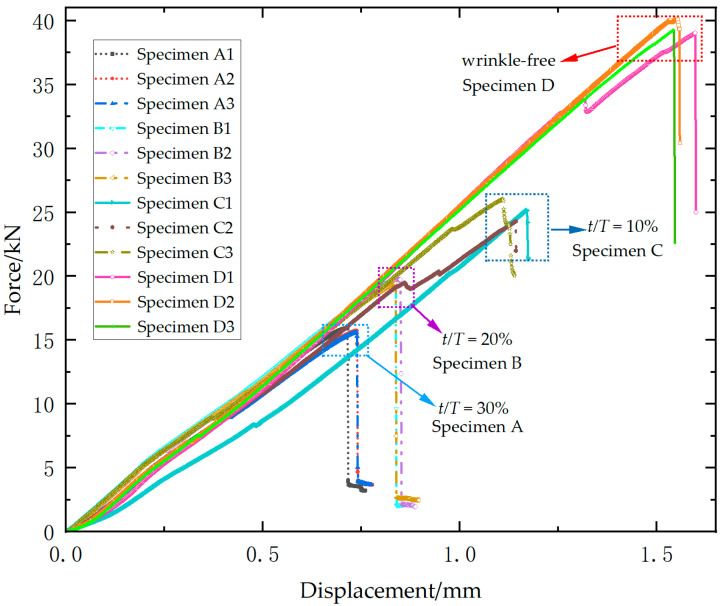
Experimental load–displacement curves of Laminate 1.

Figure 4 presents the load–displacement curves for all specimens of Laminate 1. A significant decline in ultimate load and slight reduction in stiffness is observed with increasing wrinkle amplitude-to-thickness ratio. Relative to wrinkle-free specimens, the mean ultimate loads show reductions of 36.2%, 50.6%, and 60.0% for three types of wrinkled specimens (10%, 20%, 30% *t/T*). All load–displacement curves exhibit pronounced linear characteristics, indicating that the specimens failed in a brittle manner prior to fracture. 

**Figure 5:**
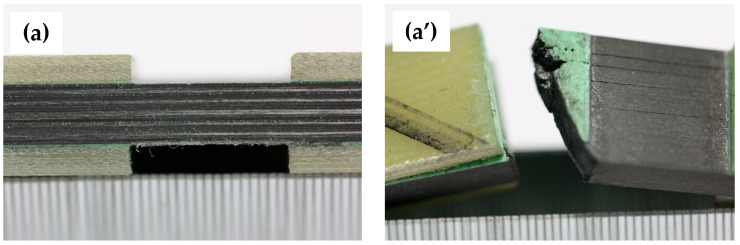

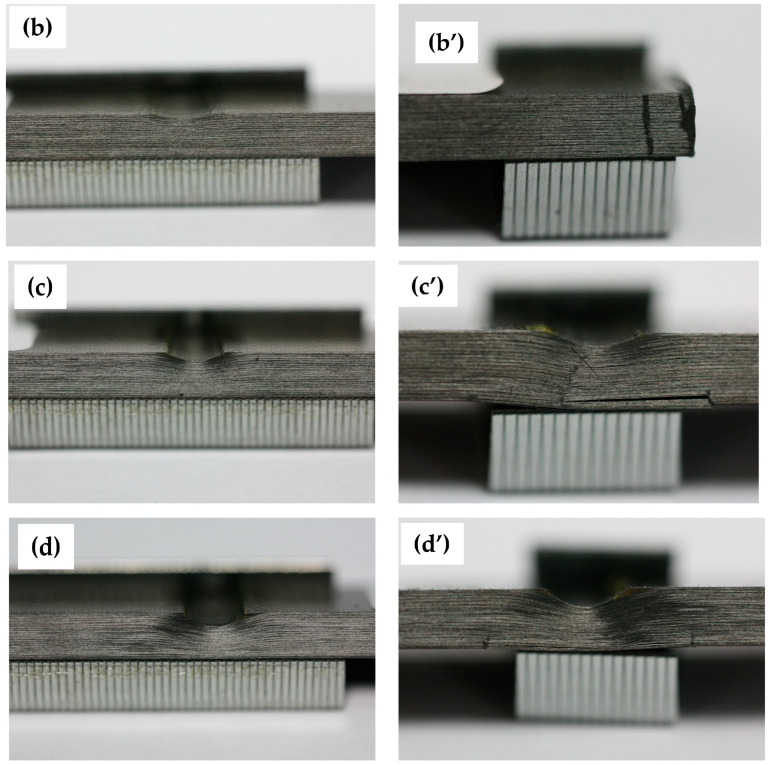
Failure of Laminate 1 with different wrinkle amplitude-to-thickness ratios: (**a**–**d**) Specimen of wrinkle-free, 10%, 20%, and 30% ratio before testing; (**a’**–**d’**) Specimen of wrinkle-free, 10%, 20%, and 30% ratio after testing.

Observations indicate that the failure mode remains consistent among specimens of Laminate 1 with different wrinkle severity within each test group. Accordingly, Figure 5 demonstrates the failure state of a representative specimen from each group. Both wrinkle-free and 10% *t/T* specimens undergo compressive failure dominated by fiber and matrix fracture (see Figure 5a’,b’), with similar ply-level failure modes, indicating negligible impact of mild out-of-plane wrinkles on the failure patterns of 0° laminate. The 20% *t/T* specimens exhibit delamination failure in addition to compressive failure in all plies (see Figure 5c’), while failure mode of the 30% *t/T* specimen is dominated by delamination, accompanied by buckling deformation toward the wrinkle side (see Figure 5d’).

**Figure 6:**
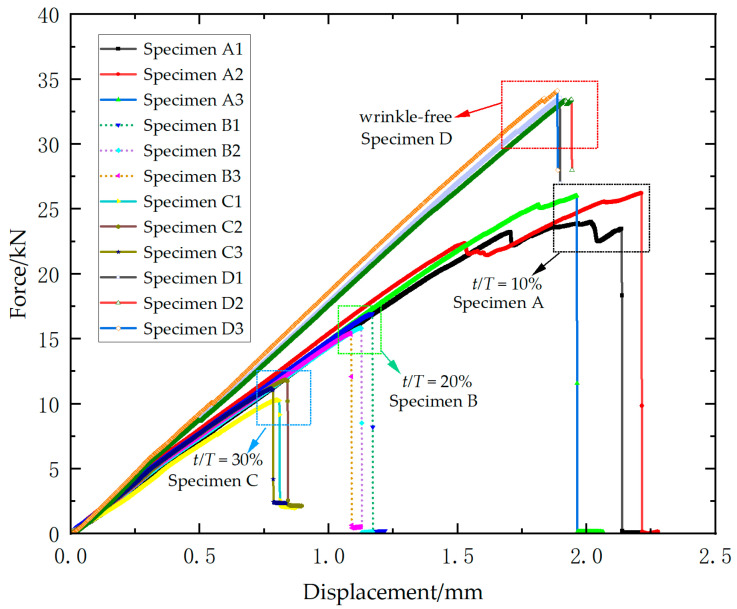
Experimental load–displacement curves of Laminate 2.

Figure 6 presents the load–displacement curves for all specimens of Laminate 2. Ultimate loads decline significantly with increasing wrinkle amplitude-to-thickness ratio, while wrinkled specimens exhibit marked stiffness reduction compared to wrinkle-free counterparts. However, specimens with different wrinkle severity ratios exhibit negligible variation in stiffness properties. For the wrinkle-free specimens, the ultimate loads are 33.6 kN, 33.5 kN, and 34.1 kN, yielding an average of 33.7 kN (standard deviation = 0.3 kN). An average ultimate load of 25.6 kN (standard deviation = 0.9 kN) is recorded for 10% ratio specimens, with individual values of 24.5 kN, 26.2 kN, and 26.0 kN. The 20% ratio group exhibit ultimate loads of 16.9 kN, 15.8 kN, and 15.4 kN, with an average of 16.0 kN (standard deviation = 0.8 kN). For the 30% ratio group, the ultimate loads are 10.2 kN, 11.8 kN, and 11.1 kN, resulting in an average of 11.0 kN (standard deviation = 0.8 kN). Relative to wrinkle-free specimens, the mean ultimate loads show reductions of 24.0%, 52.5% and 67.4% for three types of wrinkled specimens (10%, 20%, 30%). Specimens with 10% ratio exhibit progressive stiffness degradation and load fluctuations near failure, evidencing distributed damage accumulation. In contrast, specimens with higher ratios (20% and 30%) display brittle failure characterized by abrupt post-peak load drops.

**Figure 7:**
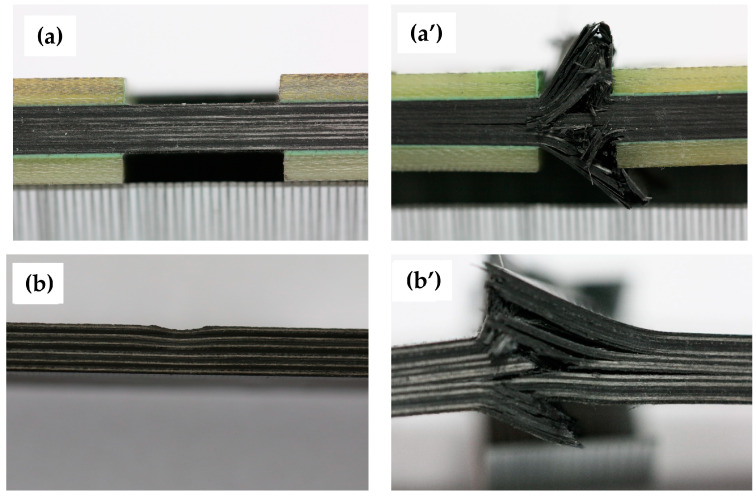

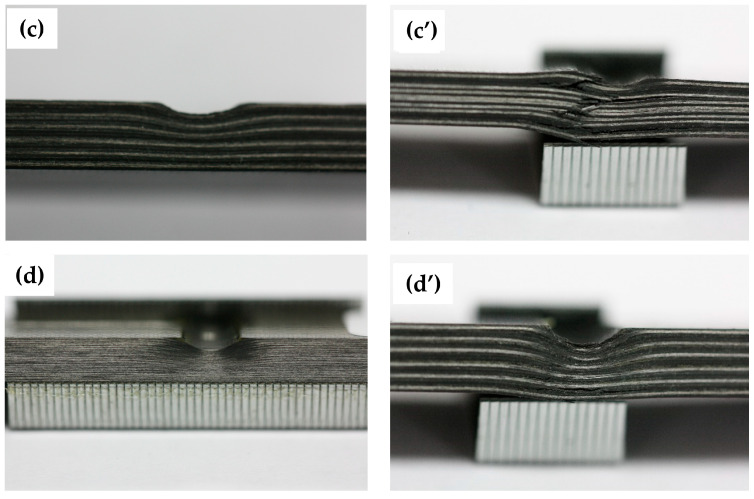
Failure of Laminate 2 with different wrinkle amplitude-to-thickness ratios: (**a**–**d**) Specimen of wrinkle-free, 10%, 20%, and 30% *t/T* before testing; (**a’**–**d’**) Specimen of wrinkle-free, 10%, 20%, and 30% –T after testing.

Similarly, observations on Laminate 2 indicate a likewise consistent failure mode across specimens of varying wrinkle severity within each test group, with representative failure states shown in Figure 7. Experimental results demonstrate compressive failure in both wrinkle-free and 10% *t/T* specimens, characterized by predominant fiber and matrix fracture (see Figure 7a’,b’). The fracture surfaces of the both specimens are highly irregular, attributed to variations in ply damage sequence and severity across different ply orientations. This indicates minimal influence of 10% fiber waviness on the failure mode. Additionally, the specimen of 20% ratio exhibited delamination failure alongside compressive failure in all 0° plies (see Figure 7c’). The 30% *t/T* specimen displayed the least severe failure, with delamination damage primarily occurring in the top and bottom plies, accompanied by buckling deformation toward the wrinkle region (see Figure 7d’).

## 4. Simulations

**Figure 8:**
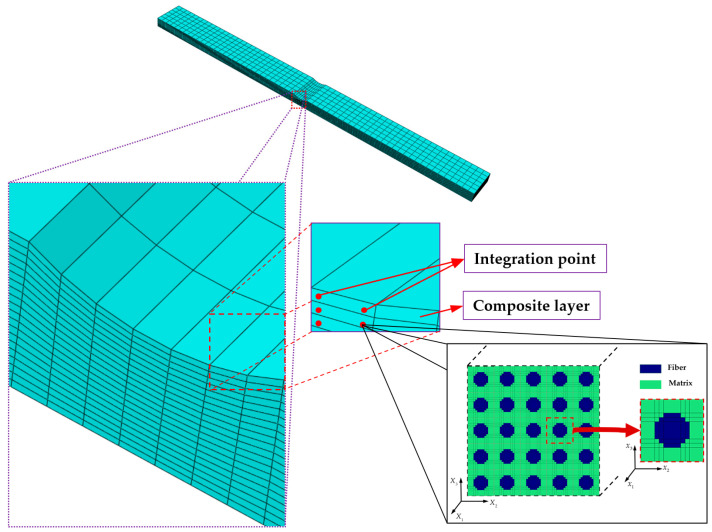
FE mesh of the wrinkled laminate and the GMC model.

The wrinkled laminate FE mesh is presented in Figure 8, wherein GMC is called at each integration point of the composite ply within the Abaqus 2022 FEM. The 3D element type C3D8R is selected for the FEM. The transverse mesh size of the laminate is set to 0.6 mm. The longitudinal mesh size in the non-wrinkled region is 1.5 mm, while the wrinkled region is discretized with a refined longitudinal mesh size of 0.4 mm. Moreover, each composite ply is discretized with one element layer through the thickness. To investigate the mechanical response comprehensively, one end of the specimen is fully constrained, whereas a prescribed displacement load is applied to the opposite end. Specifically, a reference point is positioned at the geometric centroid of the loading surface to ensure uniform load distribution across end-face nodes. A multi-point constraint (MPC) is then employed to kinematically couple this reference point with all nodes on the loading surface, enabling consistent force transfer and minimizing edge effects. The axial displacement load in the FEM is subsequently applied to the reference point, which not only standardizes the loading protocol but also streamlines the monitoring and extraction of load–displacement history data during simulation.

The unidirectional lamina elastic modulus and strength parameters provided by vendors are shown in Table 2 and Table 3. The microscopic level fiber and matrix material parameters from vendors are adjusted using the GMC model, as presented in Table 4 and Table 5.

## 5. Results and Discussion

**Figure 9:**
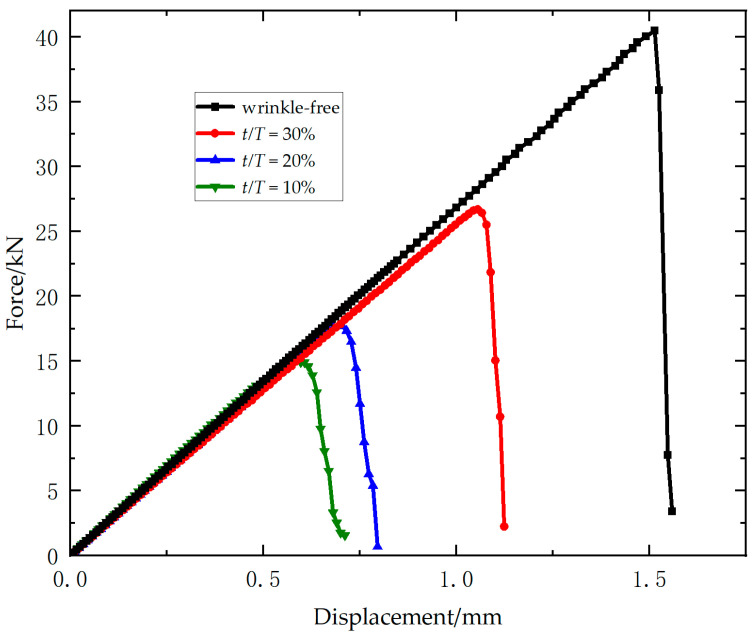
Simulated load–displacement curves of Laminate 1.

Figure 9 presents the load–displacement curves of Laminate 1 with four wrinkle severity levels obtained from multiscale simulations. All curves exhibit linear elastic behavior. Compared to the wrinkle-free model, wrinkled models show a marginal reduction in stiffness, while stiffness values remain consistent across different wrinkle severities. Progressive increases in wrinkle severity result in significant decreases in failure load and failure displacement, demonstrating the detrimental effect of wrinkles on structural strength. The simulated ultimate loads for the four wrinkled models are 40.5 kN, 26.4 kN, 17.9 kN, and 14.9 kN, with relative errors of 2.6%, 4.8%, 8.7%, and 5.4% compared to experimental data, respectively.

**Figure 10:**
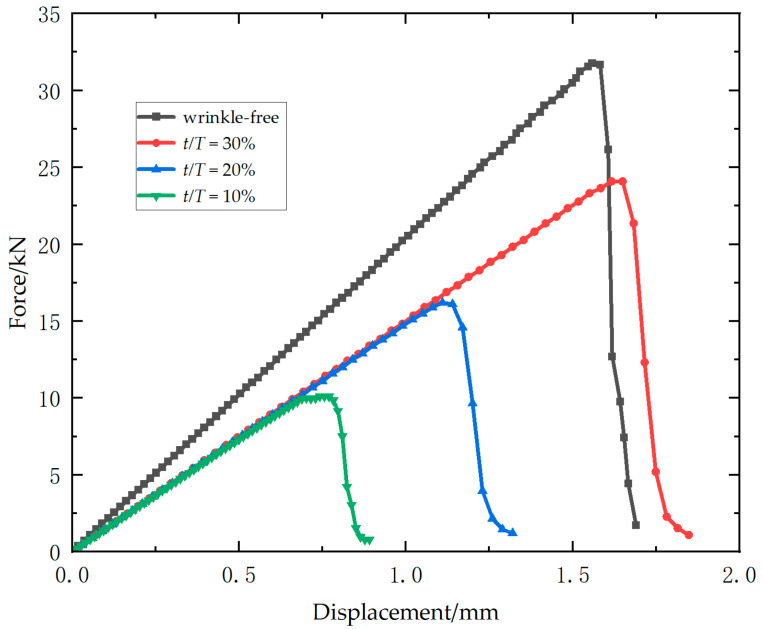
Simulated load–displacement curves of Laminate 2.

Figure 10 presents the load–displacement curves of four composite models with varying wrinkle severities in Laminate 2, derived from multiscale simulations. The wrinkled models exhibit nearly identical stiffness values, which are significantly lower (approximately 25% reduction) than that of the wrinkle-free model. Owing to this stiffness degradation, the 10% wrinkled model achieves a marginally higher failure displacement compared to the wrinkle-free counterpart. In the initial stage of the curves, all these four models exhibit nearly identical initial stiffness and follow linear elastic behavior. As the specimens with 20% and 30% *t/T* approach their ultimate loads, slight nonlinear stiffness degradation occurs, which is attributed to the out-of-plane buckling near the wrinkled side under compressive load. The simulation results exhibit a more progressive reduction characteristic compared to the corresponding experimental load–displacement curve, which can be attributed to the constraints in the simulation model that fix one end without accounting for the fixtures used in the experiment, resulting in a smaller constrained region in the simulation than in the test. Conversely, the 10% *t/T* specimen shows a less pronounced stiffness decline near its ultimate load, primarily characterized by brittle fracture failure. The simulated ultimate loads for the four specimens are 31.8 kN, 24.1 kN, 16.2 kN, and 10.1 kN, respectively, with relative error of 5.6%, 5.8%, 1.5%, and 8.8% compared to the experimental results.

**Figure 11:**
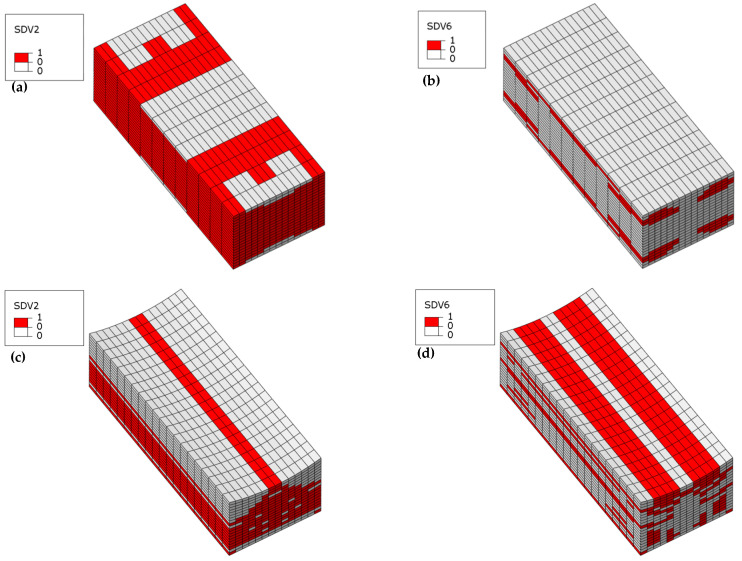

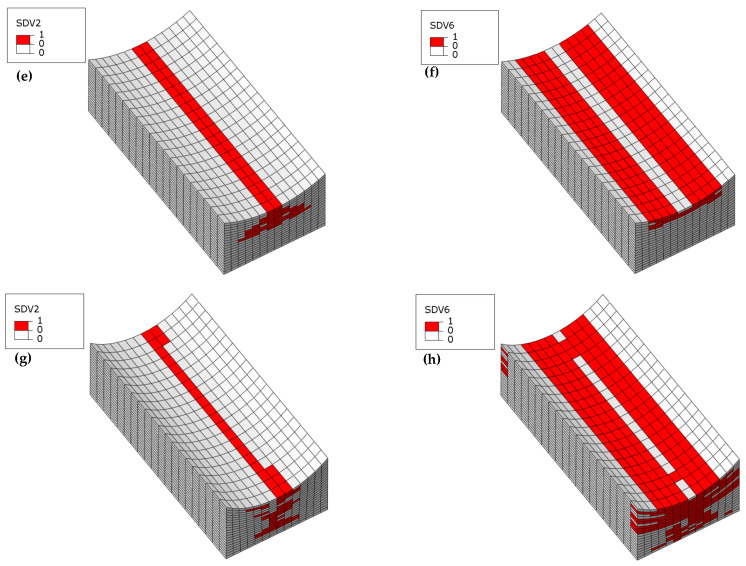
Simulated failure contour plot of Laminate 1: (**a**) Fiber compressive failure of wrinkle-free model; (**b**) Interlaminar compressive failure of wrinkle-free model; (**c**) Fiber compressive failure of 10% *t/T* model; (**d**) Interlaminar compressive failure of 10% *t/T* model; (**e**) Fiber compressive failure of 20% *t/T* model; (**f**) Interlaminar compressive failure of 20% *t/T* model; (**g**) Fiber compressive failure of 30% *t/T* model; (**h**) Interlaminar compressive failure of 30% *t/T* model.

In Laminate 1, failures under ultimate load show predominance of fiber compressive failure (SDV2) and compressive delamination (SDV6), with negligible contributions from other modes. Consequently, Figure 11 exclusively visualizes these failure types across four fiber-waviness models. The wrinkle-free specimen develops extensive fiber compressive failure throughout all plies (see Figure 11a), whereas compressive delamination localizes near outer surfaces (thickness direction, see Figure 11b). For wrinkled specimens (Figure 11c,e,g), fiber compressive failure concentrates at concave-side wrinkle troughs, with affected areas contracting as wrinkle severity escalates. Conversely, compressive delamination intensifies around these troughs and expands markedly with increased severity (Figure 11d,f,h). As the severity of wrinkles increases, the compressive failure region gradually concentrates in the plies near the fiber wrinkles. This confirms that wrinkle existence and severity critically reconfigure failure modes and spatial damage distributions in composite laminates.

**Figure 12:**
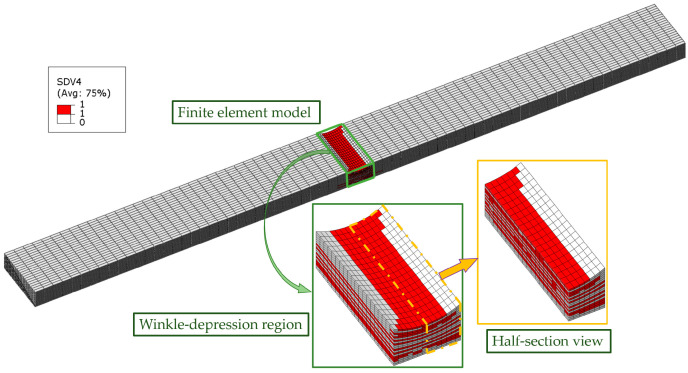
Half-sectional view of failure contour plot.

Failure locations in Laminate 2 concentrate primarily within wrinkled regions. Given that each ply corresponds to one element layer in the thickness-direction mesh, a half-section view of the wrinkle zone in the finite element model is adopted to visualize failure states for enhanced clarity. For example, Figure 12 displays the matrix compressive failure contour plot for the 20% *t/T* specimen, which can effectively visualize the damage state on the vertical cross-section of each ply. Henceforth, only half-section views will be presented and discussed, as they provide sufficiently comprehensive failure information.

**Figure 13:**
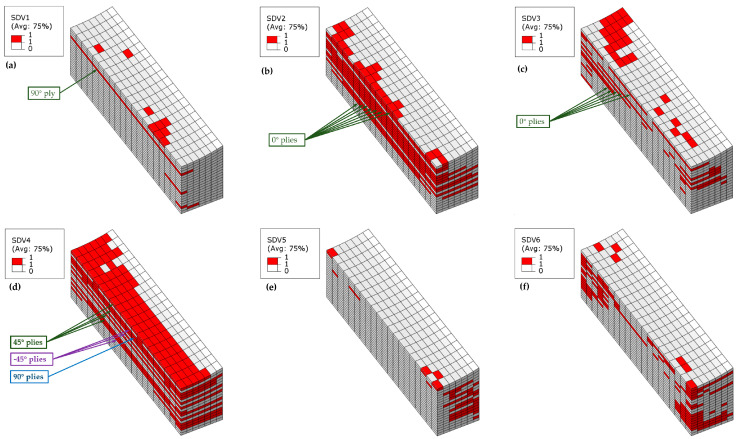
Simulated failure contour plot of Laminate 2 with 10% *t*/*T*: (**a**) Fiber tensile failure; (**b**) Fiber compressive failure; (**c**) Matrix tensile failure; (**d**) Matrix compressive failure; (**e**) Interlaminar tensile failure; (**f**) Interlaminar compressive failure.

The failure contour plots of the wrinkle-free model and the 10% *t/T* model are similar; therefore, the failure contour plots at ultimate load are presented starting from the 10% *t/T* model, as shown in Figure 13. Fiber tensile failure primarily occurs in the 90° plies on the upper side of the model (see Figure 13a), where compressive axial loading induces transverse tensile strain due to Poisson’s ratio effects. Severe fiber compressive failure dominates most 0° plies (see Figure 13b), while off-axis plies (45°, −45°, 90°) exhibit extensive matrix compressive failure (see Figure 13d), which collectively drive the abrupt loss of load-carrying capacity. Some 0° plies on the wrinkled side are accompanied by matrix tensile failure (see Figure 13c), likely caused by bending-induced tensile stresses. Delamination-related failures in the thickness direction are minimal and confined to edge elements along the width (see Figure 13e,f). The bias of matrix compressive failure and fiber kinking toward the wrinkle side indicates that compressive loading triggers out-of-plane bending deformation due to thickness-direction stiffness asymmetry. This bending amplifies stress concentrations in the wrinkle vicinity, coupling with progressive damage to dictate the final failure pattern.

**Figure 14:**
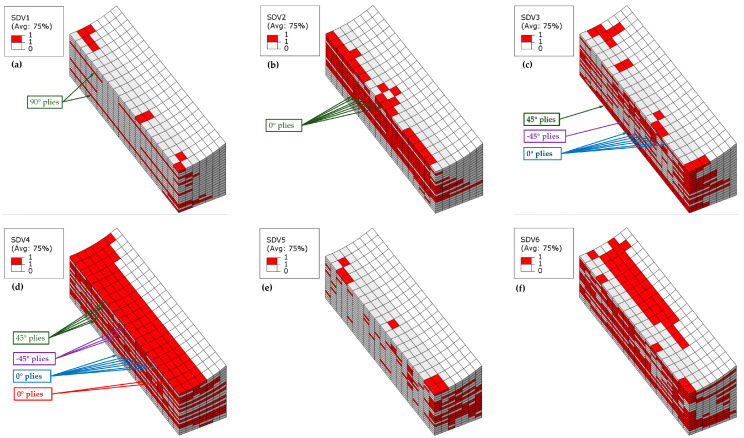
Simulated failure contour plot of Laminate 2 with 20% *t*/*T*: (**a**) Fiber tensile failure; (**b**) Fiber compressive failure; (**c**) Matrix tensile failure; (**d**) Matrix compressive failure; (**e**) Interlaminar tensile failure; (**f**) Interlaminar compressive failure.

Figure 14 displays the failure contour plot of the 20% *t*/*T* model at ultimate load. Fiber tensile failure primarily localizes in the two 90° plies (see Figure 14a), driven by transverse tensile stresses resulting from compressive loading and Poisson’s ratio effects. Severe fiber compressive failure dominates most 0° plies (see Figure 14b), while off-axis plies exhibit extensive through-thickness matrix compressive failure (see Figure 14d), with the matrix tensile damage extending to a greater number of bottom plies compared to the 10% *t*/*T* model (see Figure 14c). Tensile delamination-related failures in the thickness direction remain minimal and are confined to edge elements along the width (see Figure 14e). However, compressive delamination failures become widespread, accompanied by pronounced matrix tensile cracking in the bottom plies (see Figure 14f). The increased wrinkle amplitude amplifies both delamination propagation and bottom ply damage, highlighting the sensitivity of failure modes to geometric imperfections under compressive loading.

**Figure 15:**
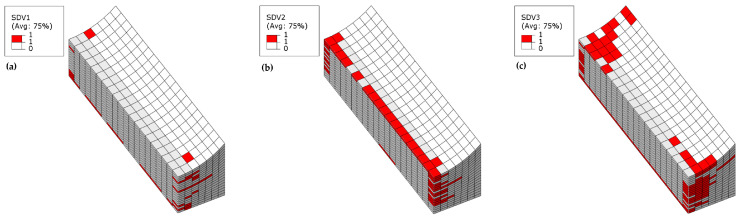

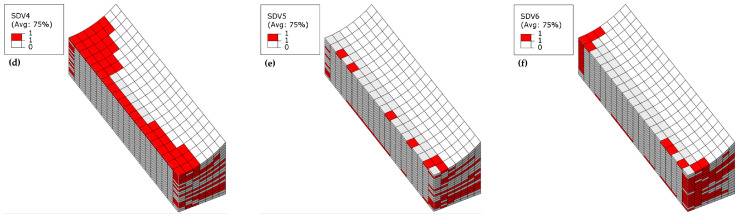
Simulated failure contour plot of Laminate 2 with 30% *t*/*T*: (**a**) Fiber tensile failure; (**b**) Fiber compressive failure; (**c**) Matrix tensile failure; (**d**) Matrix compressive failure; (**e**) Interlaminar tensile failure; (**f**) Interlaminar compressive failure.

Figure 15 shows the failure contour plot of the 30% *t*/*T* model at ultimate load. A significant reduction in load-carrying capacity is observed, with damage primarily characterized by localized fiber and matrix compressive failures in the top plies and delamination failures concentrated in edge elements along the width direction (see Figure 15b,d). Only a limited number of elements near the transverse edges exhibit fiber and matrix tensile failure, with no evidence of damage propagation completely across the transverse direction (as shown in Figure 15a,c). The extent of delamination damage is generally consistent with the tensile failure zone, though the delaminated area is slightly more extensive (see Figure 15e,f). Compared to models with lower *t*/*T* ratios, the drastic reduction in ultimate load highlights that the abrupt load drop is governed by geometric instability under compressive loading, the model undergoes pronounced out-of-plane buckling deformation toward the wrinkle region. This buckling amplifies stress concentrations in the wrinkle vicinity, leading to premature failure dominated by surface ply damage rather than progressive internal ply degradation.

## 6. Conclusions

Through compression tests and multiscale simulations on composite laminates with wrinkle amplitude-to-thickness ratios (*t*/*T*) of wrinkle-free, 10%, 20% and 30%, it is demonstrated that increasing *t*/*T* significantly reduces the load-carrying capacity. The average ultimate loads for Laminate 1 under the four severity ratios are 39.5 kN, 25.2 kN, 19.5 kN, and 15.8 kN, representing reductions of 36.2%, 50.6%, and 60.0% for the three wrinkled configurations compared to the wrinkle-free specimens. Similarly, for Laminate 2, the corresponding average ultimate loads are 33.7 kN, 25.6 kN, 16.0 kN, and 11.0 kN. Relative to the wrinkle-free specimens, the mean ultimate loads show reductions of 24.0%, 52.5%, and 67.4% for the three wrinkled types. The multiaxial progressive damage model, based on the Generalized Method of Cells (GMC), effectively captures damage modes in the wrinkled region and predicts ultimate loads. Key failure mechanisms include fiber compressive failure, matrix compressive crushing, and through-thickness compressive delamination under compression. As *t*/*T* increases, geometric imperfections induce localized stiffness reduction, promoting pronounced out-of-plane buckling deformation toward the wrinkle. This buckling redistributes stresses: compressive failure intensifies in upper plies near the wrinkle, while bending-induced tensile stresses drive failure in bottom plies. The close agreement between experimental and simulated failure modes, ultimate loads, and load–displacement curves validates the proposed multiscale framework’s reliability and feasibility for predicting wrinkle-driven composite failure.

## Figures and Tables

**Table 1:** Sudden degradation rules of constituent material property.

Failure Mode	Sudden Degradation Methodology
Subcell failure in the *x*_1_-direction	$E_{11}^{d}=10^{-4}\times E_{11}$
Subcell failure in the *x*_2_-direction	$E_{22}^{d}=10^{-4}\times E_{22},\;G_{12}^{d}=10^{-4}\times G_{12},\;G_{23}^{d}=10^{-4}\times G_{23}$
Subcell failure in the *x*_3_-direction	$E_{33}^{d}=10^{-4}\times E_{33},\;G_{12}^{d}=10^{-4}\times G_{12},\;G_{23}^{d}=10^{-4}\times G_{23}$

**Table 2:** Mechanical properties of the unidirectional lamina.

E_1_/GPa	E_2_/GPa	E_3_/GPa	G_12_/GPa	G_13_/GPa	G_23_/GPa	ν_12_	ν_13_	ν_23_
145	8.5	8.5	4.5	4.5	4.5	0.3	0.3	0.35

**Table 3:** Strength properties of the unidirectional lamina.

*X_T_*/MPa	*X_C_*/MPa	*Y_T_*/MPa	*Y_C_*/MPa	*Z_T_*/MPa	*Z_C_*/MPa	*S*_12_/MPa	*S*_13_/MPa	*S*_23_/MPa
2700	1450	90	200	90	200	110	110	110

**Table 4:** Elastic properties of the composite constituents.

Fiber Properties	Value	Matrix Properties	Value
Longitudinal modulus *E*_*f*11_	270 GPa	Modulus *E_m_*	2000 MPa
Transverse modulus *E*_*f*22_	90 GPa	Poisson's ratio *v_m_*	0.35
Major Poisson's ratio *v*_*f*12_	0.23	Shear modulus *G_m_*	2300 MPa
Transverse shear modulus *v*_*f*23_	0.45	Fiber volume fraction *v_f_*	0.52
Transverse shear modulus *v*_*f*23_	0.45	Fiber volume fraction *v_f_*	0.52

**Table 5:** Strengths of the composite constituents.

Strength	Value (MPa)
*X_T_*	tensile strength of the fiber 7200
*X* *_C_*	compressive strength of the fiber 2900
*X_T_*,*Y_T_*, *Z_T_*	tensile strength of the matrix 49
*X_C_*, *Y_C_*, *Z_C_*	compressive strength of the matrix 124

## Data Availability

The original contributions presented in this study are included in the article. Further inquiries can be directed to the corresponding authors.
